# RELATIONAL COLLABORATION BEYOND PROFESSIONAL TEAMS: NEGOTIATING HYBRID EXPERTISE WITH INFORMAL CAREGIVERS

**DOI:** 10.1093/geroni/igae098.3334

**Published:** 2024-12-31

**Authors:** Laura Ginoux, Kirstie McAllum

**Affiliations:** Université de Montréal, Montréal, Quebec, Canada; Université de Montréal, Montréal, Quebec, Canada

## Abstract

When conceptualizing collaboration in the context of health and social care, most definitions privilege interactions among members of the interprofessional team. To achieve joint goals and solve problems more effectively, these professionals must combine and integrate their disciplinary knowledge with that of others, including those outside the immediate healthcare team. Over the last three decades, the health communication literature has insisted on the need for patient-centered healthcare practices. Patient-centeredness includes appreciation for and inclusion of patients’ experiential knowledge (based on their daily experiences with health conditions). We note that the literature neglects the idea of experiential expertise of informal caregivers (ICG). Yet, ICGs are precious aids and mediators between the older adults they care for and healthcare workers (HW). Unfortunately, ICGs are not always included in decision-making processes, and hierarchies of knowledge remain within relationships with HWs. Following a literature review, our research highlights the need to develop a relational vision of collaboration that would enable interprofessional teams to consider ICGs as part of the team, enhance the quality of interactions, and, therefore, improve the quality of care provided to older adults. The objectives of this paper are threefold. First, we define our theoretical model of relational collaboration. Second, we explain why developing relational collaboration between ICGs and HWs benefits teams as well as older adults. Third, we contend that nurturing the relational dimensions of collaboration (respect, cultural humility, empathy, compassion, and trust) enables ICGs and HWs to negotiate a hybrid form of expertise that integrates medical and experiential knowledge.

